# FeinPhone: Low-cost Smartphone Camera-based 2D Particulate Matter Sensor

**DOI:** 10.3390/s19030749

**Published:** 2019-02-12

**Authors:** Matthias Budde, Simon Leiner, Marcel Köpke, Johannes Riesterer, Till Riedel, Michael Beigl

**Affiliations:** Karlsruhe Institute of Technology (KIT), TECO/Pervasive Computing Systems, Karlsruhe 76131, Germany; leiner@teco.edu (S.L.); koepke@teco.edu (M.K.); riesterer@teco.edu (J.R.); riedel@teco.edu (T.R.); beigl@teco.edu (M.B.)

**Keywords:** mobile sensing, PM, ubiquitous computing, atmospheric dust, Particulate Matter, low-cost sensor

## Abstract

Precise, location-specific fine dust measurement is central for the assessment of urban air quality. Classic measurement approaches require dedicated hardware, of which professional equipment is still prohibitively expensive (>10k$) for dense measurements, and inexpensive sensors do not meet accuracy demands. As a step towards filling this gap, we propose *FeinPhone*, a phone-based fine dust measurement system that uses camera and flashlight functions that are readily available on today’s off-the-shelf smart phones. We introduce a cost-effective passive hardware add-on together with a novel counting approach based on light-scattering particle sensors. Since our approach features a 2D sensor (the camera) instead of a single photodiode, we can employ it to capture the scatter traces from individual particles rather than just retaining a light intensity sum signal as in simple photometers. This is a more direct way of assessing the particle count, it is robust against side effects, e.g., from camera image compression, and enables gaining information on the size spectrum of the particles. Our proof-of-concept evaluation comparing several *FeinPhone* sensors with data from a high-quality APS/SMPS (Aerodynamic Particle Sizer/Scanning Mobility Particle Sizer) reference device at the World Calibration Center for Aerosol Physics shows that the collected data shows excellent correlation with the inhalable coarse fraction of fine dust particles (r > 0.9) and can successfully capture its levels under realistic conditions.

## 1. Introduction

Location-specific sensing of atmospheric particles plays an important role in capturing the dynamics of urban air quality as well as quantifying individual exposure. Numerous studies have revealed severe health effects of Particulate Matter (PM) pollution in the past [1,2,3]. The rise of real-time capable particle sensors has enabled a paradigm shift towards distributed measurements with high spatial and temporal resolution [4], with the potential to augment existing monitoring systems and enable citizen science environmental monitoring.

Existing Particulate Matter (PM) sensing approaches, however, cannot meet the demands of these new sensing scenarios. PM sensing today generally requires dedicated devices. Professional equipment is too costly to be deployed on a large scale and inexpensive sensors are not yet ready for applications that require high accuracy [5,6]. Current low-cost laser-scattering sensors, such as the *Nova Fitness SDS011* [7] may show satisfactory correlation under certain conditions, but are, for example, generally unable to adequately capture PM${}_{10}$ levels well, especially under changing particle size distributions [8,9].

This paper presents a novel way of optically counting suspended particles which is based on the light-scattering (*nephelometry*) measurement approach. By using a camera instead of a single-pixel photodiode as light receptor in combination with image processing algorithms, our approach can capture and count the light scatter traces from individual particles. This yields additional information on the size spectrum of the observed particles.

As practical implementation of our approach, we present *FeinPhone*, a phone-based fine dust measurement system with a ultra-low-cost passive hardware add-on that uses camera and flashlight functions that are readily available on today’s off-the-shelf smartphones.

## 2. Related Work

A wide range of measurement approaches has been applied to the measurement of Particulate Matter (PM). In recent years, handheld fine dust monitoring has increasingly been studied. Most works rely on optical measurement approaches, as they are currently the most suitable for low-cost measurements. Several authors have used *light-scattering* dust sensors for mobile and/or distributed fine dust measurements [10,11]. However, all of these approaches require dedicated devices.

In *direct imaging*, an aerosol flows in between a light source and a camera. Passing particles occlude the light, making it possible to detect them through image analysis. The *CAMSIZER 2X* is a product that enables measurement as well as size and shape segregation of particles between 0.8 μm and 8 mm. A drawback of the system is that it takes very high concentrations for reliable measurements [12].

Another camera-based approach is *dust deposition imaging* [13]. The detection simply is periodically taking an image from a high-resolution CMOS or CCD image (camera) sensor, which is installed at a 45 ${}^{\circ }$ tilt. Through illumination with a uniform light source, deposited particles occlude individual pixels that can in turn be counted by differentially comparing images over time. Since it relies on gravitational sedimentation, this technique is aimed at indoor measurement as it can only measure particles in turbulence-free environments. Because of relatively low deposition rates of small particles, sampling intervals need to be long compared to other methods and only the coarse fraction can be detected. The operation principle has been successfully evaluated in the Vatican museums in Rome [14].

A system to measure Black Carbon (BC) with cellphones was presented by Ramanathan et al. [15]. The *aethalometry*-based approach involves BC aerosol collection on a quartz filter, the coloration of which is then captured by the phone’s camera and transmitted to an analytics component for real-time evaluation.

Another interesting approach to measuring atmospheric dust in Participatory Sensing scenarios has been presented by the *Air Visibility Monitoring* [16] respectively the *iSPEX* [17] projects. In the former, people use their camera phones to take pictures of the sky and upload them to a central database. There, from the image luminance, the location and phone sensor data (e.g., orientation), the particle pollution is estimated. Cloudy skies and indoor environments present clear limitations to this approach. The *iSPEX* system makes use of a passive spectropolarimetric clip-on module for the iPhone. It has been successfully used in single-day measurement campaigns on a Participatory Sensing scale.

Other efforts to enable particulate matter sensing with smartphones have been made in the past, among them approaches that aim at developing small sensors that can actually be integrated into the casing of a smartphone: Carminati et al. presented the design of a capacitive particle sensor that has the potential to be micro-fabricated and embedded into phones [18]. In a different approach, Doering et al. enabled direct measurement of the mass concentration of particles with an air-microfluidic micromechanical (MEMS) design [19]. These developments both show interesting approaches as well as promising performance. However, there may be one issue with the general approach of miniaturizing sensors that far. This is not a question of detection principle, rather purely one of statistics: The smaller the detector volume, i.e., the amount of air that can be sampled at a time, the more consecutive measurements need to be taken in order to make a statistically reliable statement concerning the mean concentration. This has implications for air flow and measurement frequency requirements.

In previous work, we have ourselves demonstrated the feasibility of direct smartphone-based PM measurement by using the flash LED and camera of a smartphone as active components of a clip-on nephelometry fine dust sensor [20,21]. Other work has shown that a phone’s camera and flash LED can be leveraged beyond taking photos, e.g., to measure physiological parameters [22], such as the heart rate [23] or SpO${}_{2}\%$ levels [24,25], for fluorescence-based measurements with disposable optical sensor chips [26], or even turn a mobile phone into a Geiger counter [27]. However, research on mobile, low-cost and participatory sensing does recognize the need to ensure credible readings from cheap sensors [28].

This work therefore takes the clip-on light-scattering approach to the next level by changing the measurement principle to capture and count light scatter traces with a camera sensor rather than a light scattering sum signal. This approach is a more direct way of assessing the particle count, is robust against side effects, e.g., from camera image compression, and enables gaining information on the size spectrum of the detected particles.

## 3. Measurement Principle

**Figure 1:**
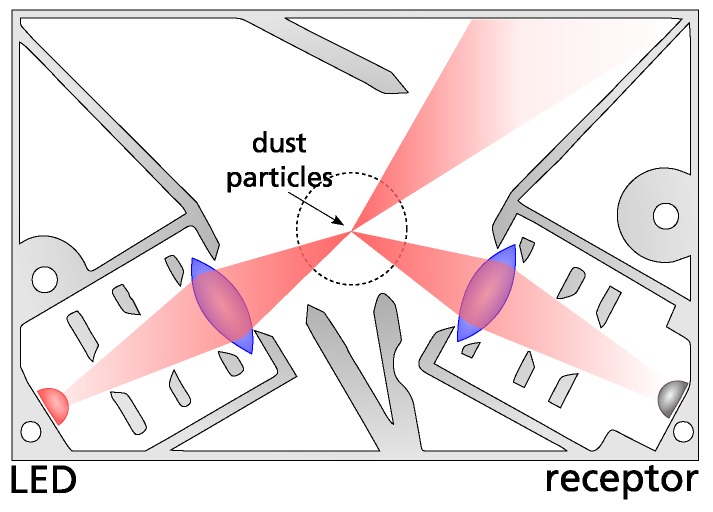
In light-scattering sensors (a.k.a. nephelometers), light is emitted into a measurement chamber. If there are particles present, the light is refracted and collected by a photodiode.

In order to count suspended atmospheric particles using a camera, we modify the established light-scattering measurement approach. In light-scattering, also known as nephelometry, the light from an LED or laser is emitted into a measurement chamber. If no particles are present, it is caught in a light trap. Otherwise, the light is scattered by the particles and detected by a photodiode. The principle is illustrated in Figure 1.

### 3.1. Light-Scatter Trace Counting

In previous research, we have demonstrated that the light scattering approach can in principle be transferred to camera phones [20]. As with other photometers, the captured brightness represents a sum signal (count, size) from any particles that are present in the measurement volume. Information on how many particles of which size are present in the sampled air is not accessible with simple nephelometers.

**Figure 2:**
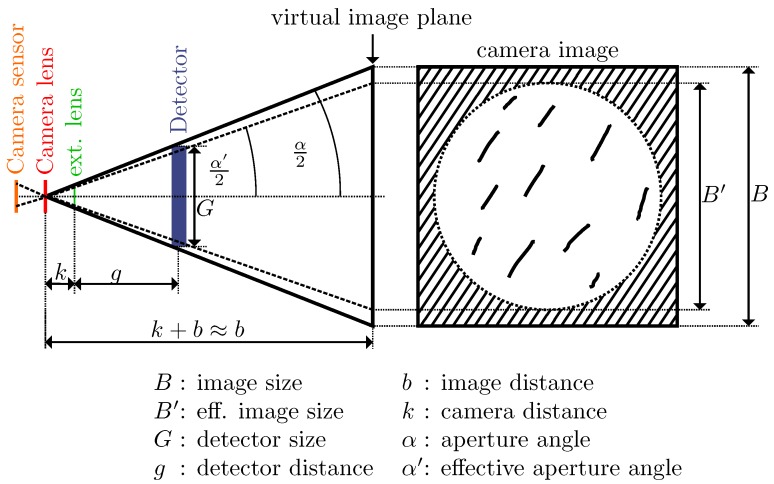
Sketch of the magnifier-based sensor principle: This detector design allows virtual images of the scatter traces that are created by individual particles traveling through the measurement volume.Sketch of the magnifier-based sensor principle

In this paper, we therefore modify this approach: We adjusted the optical system to actually see the particles move, by changing the optical layout to a magnifier-based approach, which makes use of additional information that is potentially available due to the fact that the detector is a 2D matrix sensor (camera) instead of a single pixel (photodiode). Before, all rays from the measurement chamber were collimated onto the camera chip. In the magnifier approach, the camera is no longer located at the focal point of the lens. Instead it creates a virtual image of the dust particles (see Figure 2). By this, the camera image becomes a visual representation of the inside of the detector volume. All that is required for this is the use of a single lens with large magnification.

In this way, additional information on the particle sizes can be retrieved from the camera images, while the overall brightness should still be proportional to the overall particle count. Which particles can still be seen as traces on the camera image depends on the employed magnification. In order to still be able to sample a sufficiently large air volume, the magnification should not be chosen to actually display the particles themselves, which are in the micrometer range. Instead, the virtual images will just show their scatter traces. The rationale behind this approach is as follows: Firstly, displaying individual particles is a more direct way of assessing the particle count, compared to deriving it from a sum signal. Secondly, the scattered light from individual particles is concentrated onto a smaller area, so that the sensitivity of the sensor should be higher. Lastly, individual traces have the potential to reveal further information, for instance on the size spectrum of the detected particles.

### 3.2. Optical Parameters

If the distance *g* between the lens and the detector volume is sufficiently small compared to the lenses focal length *f*, the magnifying lens creates a virtual image of the particles: The camera “sees” the particles on the virtual image plane. These are magnified by the factor

(1) $$V=\frac{b}{g}$$

In addition, the virtual image is always in focus. As long as the distance *k* between the camera and the magnifying lens is sufficiently smaller than *f*, *k* is irrelevant to the image focus. However, *k* does have an impact on the ratio of the image that the camera sees through the lens: If the lens is close to the camera, it fills the whole camera image. When *k* increases, the lens only takes up a part of the image and the environment of the lens is visible at the edges. When the total width of the image is *B*, we call the effective diameter of the image through the lens $B^{\prime }=:p\;\cdot \;B$. If *p* is too small, the entire detector volume is not visible anymore.

We want *p* to be very close to 1, so we choose *k* as small as possible.

(2) k+b≈bandk+g≈g

From Figure 2, we get:

(3) $$\frac{G}{g}=\frac{B}{b}$$

In addition, using Equation (2):

(4) $$\begin{array}{c} \tan\left(\frac{\alpha^{\prime }}{2}\right)=\frac{B^{\prime }}{2b}=\frac{B}{2pb}\underset{\left(\right)}{=}\frac{G}{2pg}\\ ⇕ \end{array}$$

(5) $$\begin{array}{c} g=\frac{G}{2p\;\cdot \;\tan(\frac{\alpha^{\prime }}{2})} \end{array}$$

The lens formula for virtual images is:

(6) $$\frac{1}{f}=\frac{1}{g}-\frac{1}{b}$$

If we combine Equations (4) and (6), we get:

(7) $$f=\frac{G\;\cdot \;b}{2\;\cdot \;p\;\cdot \;b\;\cdot \;\tan(\frac{\alpha^{\prime }}{2})-G}$$

Now, *b* can be determined:

(8) $$b=\frac{G\;\cdot \;f}{2\;\cdot \;p\;\cdot \;f\;\cdot \;\tan(\frac{\alpha^{\prime }}{2})-G}$$

Summing up, there are three free parameters: The distance between the camera and the magnifying lens *k* should be chosen to be as small as possible. In order to get an optimal magnification (see Equation (1)), the distance *g* between the magnifying lens and the detector should be as small as possible. On the other hand, construction limitations apply: *g* must be big enough so that the lens can actually be inserted into the measurement chamber and the lens does not intrude into the actual detector volume. The focal length *f* of the magnifying lens has a very small impact on the magnification *V*. However, it must be bigger than *k* and *g*, as this was our fundamental assumption.

## 4. The FeinPhone System

The *FeinPhone* sensor system realizes the proposed measurement approach on off-the-shelf camera smartphones. The system consists of two parts: A passive clip-on module inspired by low-cost light-scattering dust sensors and a signal processing algorithm to process the recorded camera images.

### 4.1. Hardware Design

**Figure 3:**
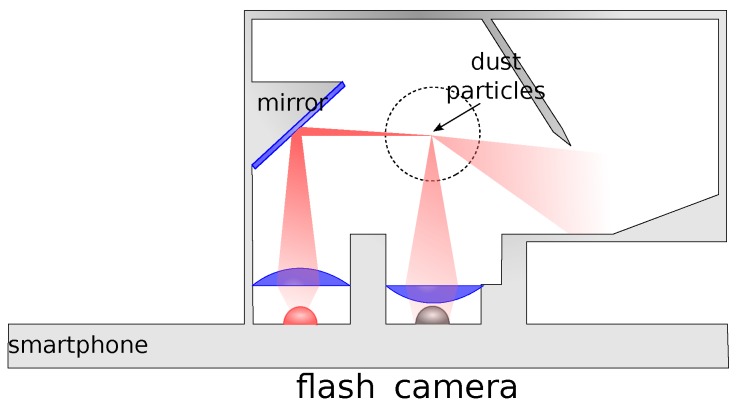
Custom light trap design with a mirror to relay the light from the flash LED into the measurement chamber.

The design of the clip-on light-scattering sensor only uses the phone’s internal camera as light receptor and flash LED as light emitter (see Figure 3). This passive design has the benefit that it can be realized at extremely low cost. Through the clip-on approach the sensor is easy to install and can be removed when it is not needed, enabling regular use of the phone’s camera.

Compared to the previous proof-of-concept approach [20], several changes were made. The past version had an insufficient light intensity in the measurement chamber, as well as impracticably large dimensions of the clip-on module. To remedy this, we designed a custom measurement chamber that employs a mirror to relay the light from the camera flash LED, instead of the optical fibre used in the previous version. The measurement chamber was developed in an iterative rapid prototyping fashion. The clip-on modules were 3D printed for quick testing. They feature lenses and mirrors as additional manually installed optical elements. We printed the sensors from black thermoplastics (PLA or ABS).

**Figure 4:**
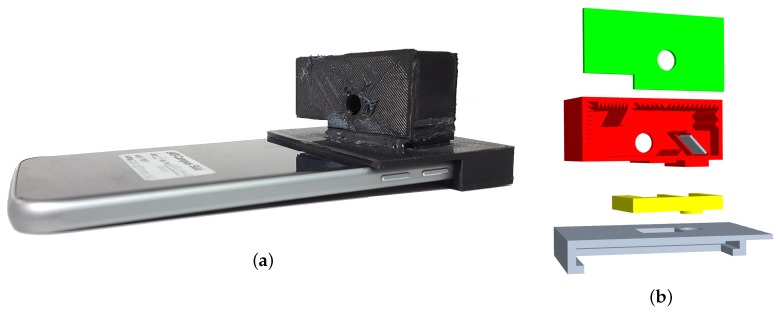
(**a**) *Galaxy S6* phone with *FeinPhone* sensor prototype. (**b**) The clip-on modules were 3D printed for rapid prototyping.

In order to increase the amount of light that is emitted into the measurement chamber, we added a semi-spherical collimator lens to attempt to parallelize the diffuse light from the flash LED. A mirror is then used to illuminate the measurement chamber. The second employed lens was chosen to realize the magnifying optical approach as described above. A prototype was designed for a *Galaxy S6* smartphone (see Figure 4).

The biggest advantage of the passive design is simply that: it is passive. This makes it ultra-low-cost and the control of the whole measurement can be implemented in software on any phone. A drawback is that the layout of the camera and the flash LED is model dependent, so the physical sensor design of the clip-on module would have to be adopted for different phone types according to their individual geometry.

### 4.2. Algorithm Design

In order to derive a particle count from the images that are recorded with the presented hardware design, an appropriate algorithm had to be developed.

#### 4.2.1. Contour Detection Particle Counting (CDPC)

**Figure 5:**
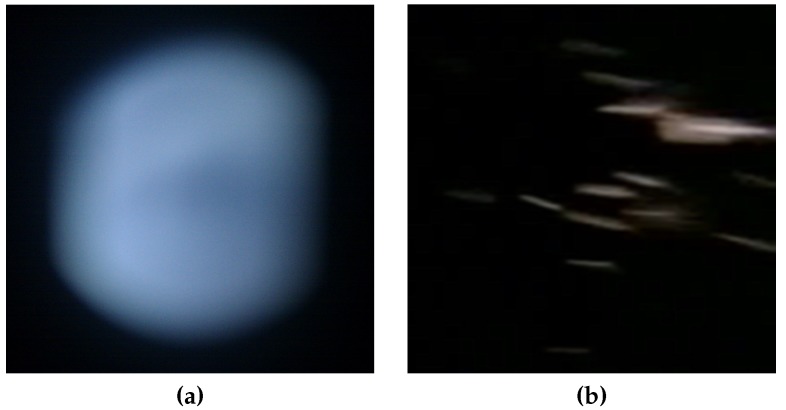
(**a**) Scatter blob [29] vs. (**b**) individual scatter traces, for illustrative purposes both at high particle concentrations.Scatter patterns: blob v. individual traces

As presented above, the images recorded through the *FeinPhone* sensor capture individual scatter traces from particles rather than a sum signal. The difference in camera images between the established light-scattering [20] and the novel scatter trace counting approach are shown in Figure 5.

In order to detect individual particles from the *FeinPhone* image data, a combination of signal processing steps and standard algorithms from the *OpenCV* computer vision library [30] was used. The complete CDPC algorithm is shown in Algorithm 1.

**Figure 6:**
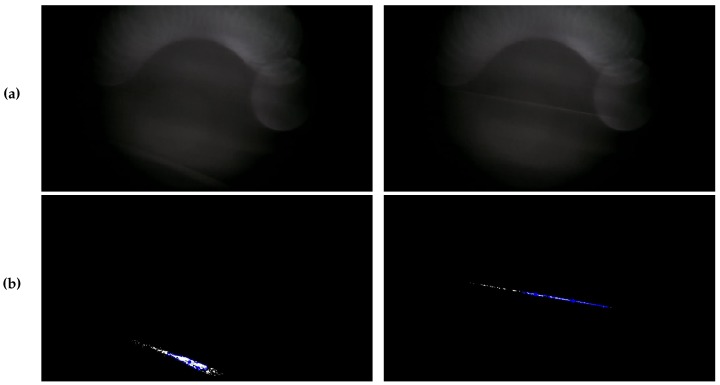
Contour Detection Particle Counting: CDPC The original recordings (**a**) undergo background subtraction, blur and binarization before a contour detection algorithm isolates continuous patches, of which all with an area exceeding a preset threshold are counted (**b**).

The algorithm takes a time series of RGB bitmap images (i.e., a video) as input. As a preprocessing step, the first five seconds of the video were cut off, since the first frames in the beginning may be affected with noise due to automatic camera adjustments, such as setting the configured focus length, etc. Subsequently, we converted the RGB video to grayscale. The first major step is processing the video with a background subtraction algorithm, since the images from our custom *FeinPhone* sensors all were affected by background illumination due to imperfect light trap design and the manual assembly of the sensors (see Figure 6). We used one of the standard background/foreground segmentation methods available in *OpenCV*: The *MOG2* subtractor is based on Gaussian Mixture Models [31,32] in order to determine and remove the static background pixels. It features shadow detection, which was disabled in our analysis since it was not required.

The so-called learning rate $r_{learn}$∈[0,1] is a parameter of the *MOG2* algorithm that specifies how fast the background model is learned, respectively updated. A value of 0 means that the background model is not updated at all, assigning a rate of $r_{learn}=1$ means that every frame, the background model is completely reinitialized from the previous frame. Since we have an almost static background, we only varied the learning rates close to 0. The parametrization is discussed in the evaluation section.

Subsequent to the background subtraction, another second was cut from the beginning of the video, as the first few frames may have still displayed some artifacts until the background model was learned. The next step of the algorithm applies a Gaussian blur filter. This is a common step in image processing before contour or edge detection, as it reduces the noise in the image, thus preventing the contour detection from falsely identifying noise as edges.

The next image processing step is to detect contours in the blurred images. For this, the findContours() function of *OpenCV* was used. It was configured to use RETR_EXTERNAL as contour retrieval mode, meaning that it did not return hierarchical contours, i.e., contours within other contours. 
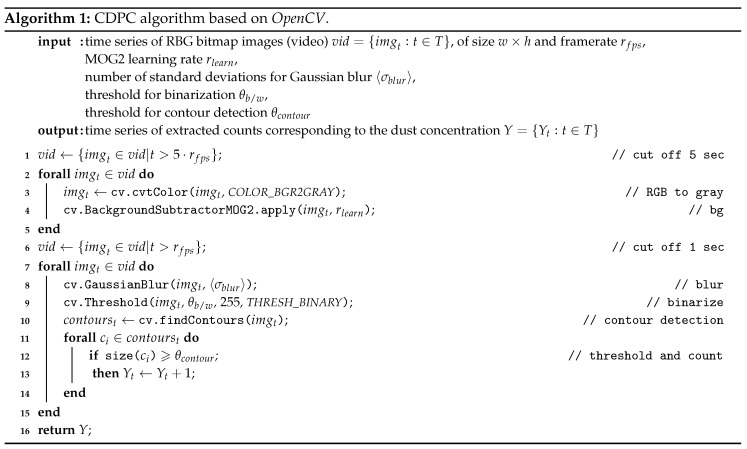


Finally, all contours are counted in each frame if their area exceeds a certain threshold. This parameter can be used to tweak the algorithm in order to differentiate between actual particles and possible miscountings due to remaining noise. Figure 6 shows two examples of the original images from our evaluation (a) and the respective images after application of the CDPC algorithm (b).

In principle, this yields a count of particles which could in turn be used to directly calculate the mass concentration for a time window, assuming a mean density and particle diameter. However, to obtain useful readings, since a mean concentration needs to be calculated for a certain period of time and to be more robust against imperfect parametrization, we decided to feed the output of the CDPC algorithm into another processing step.

#### 4.2.2. Poisson Particle Detection (PPD)

The *Poisson Particle Detection (PPD)* algorithm has been proposed by us in previous work as a simple signal reconstruction scheme for data from environmental phenomena that can be modeled as particles [29]. It was designed to reconstruct the “true” signal from noise-afflicted data solely from the Poisson noise of a signal.

The idea of the approach is very simple: the observation of a changing concentration of particles in a measurement chamber is the process of counting uniformly distributed independent events in a spacial volume. This is by definition a Spatial Poisson Process (SPP). In a Poisson process, there is signal dependent noise: The number of observed occurrences fluctuates with a standard deviation of $\sigma_{n}=\sqrt{\bar{n}}$ around its mean $\bar{n}$. From this noise a reconstruction of the mean concentration of the signal can be calculated while at the same time removing systematic measurement error [29]. The PPD algorithm is shown in Algorithm 2. 
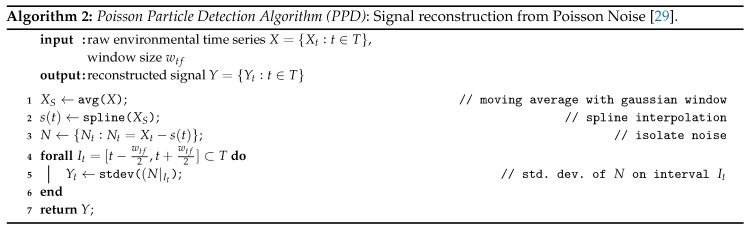


It has four steps: After a simple moving average filter is applied, a spline interpolation is constructed that represents the mean value on any point within the measurement series. After that, the noise is isolated by subtracting this approximated mean from the original raw data.

In past research, we applied the PPD algorithm directly to the sum signal of a light scattering particle sensor [29]. In this work, we combine it with the counting algorithm and the PPD algorithm. When the two algorithms are applied in succession, the CDPC delivers a particle count and the PPD smooths the discrete counts, yielding a signal corresponding to the mean concentration for time intervals given by the selected window size $w_{tf}$, while at the same time removing possible error that may be introduced by less than perfect parametrization of the CDPC.

## 5. Evaluation

In order to evaluate the smartphone-based PM sensing approach, we compared several *FeinPhone* prototypes with reference measurements in a controlled lab setting at the World Calibration Center for Aerosol Physics (WCCAP). The WCCAP is a facility at the Leibniz Institute for Tropospheric Research (TROPOS, Leipzig, Germany), that is operated in cooperation with the German Federal Environmental Agency (UBA) and the World Meteorological Organization (WMO). It conducts calibrations of physical aerosol measurement instruments as well as environmental and work place measurements of aerosols.

**Figure 7:**
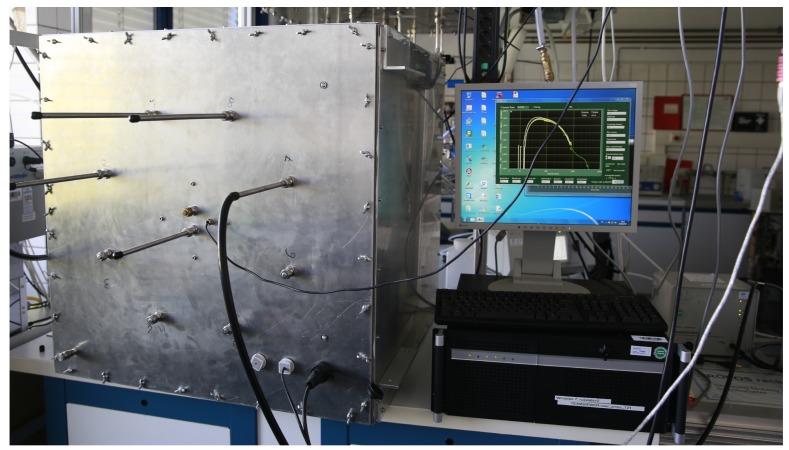
Evaluation Setup: The *FeinPhone* prototypes were placed inside an aluminum measurement chamber (**left**), into which a varying concentration of polydisperse particles was injected. A custom SMPS and a TSI APS (**right**) were used for reference measurements.

The experiment setup is depicted in Figure 7. Through the air inlet of an otherwise airtight aluminum chamber, into which five *FeinPhone* prototypes were placed, ammoniumsulfate was injected in order to create a rising concentration of polydisperse particles inside the chamber. The temperature in the chamber was 25${}^{\circ }C$ to 26${}^{\circ }C$ and relative humidity (RH) was below 15%. The air outlet was connected to two reference devices: A TSI Aerodynamic Particle Sizer (APS) Spectrometer Model 3321 [33] and a Scanning Mobility Particle Sizer (SMPS) custom made at the WCCAP [34]. The combination of these two devices enabled the measurement of 92 aerodynamic size channels between 10 nm and 20 μm. Time resolution was one reading every 4.5 min, since the SMPS samples the different channels in a time multiplex fashion, i.e., the channels are sampled one after another over the course of 4.5 min, after which all of the readings are output.

Within this time, faster dynamics cannot be captured correctly. In addition, the time by which individual readings may be off may be as a large as 9 min for the channels that are sampled first.

From the 92 channels, we calculated the three size classes PM${}_{10}$, PM${}_{2.5}$ and PM${}_{1}$ in the following way: We assumed the ammoniumsulfate particles to be spherical and homogenic with a density ρ of 1.7 g $m^{3}$. With this, the size spectrum can be converted to a geometric diameter

$$d_{geometric}=d_{aerodynamic}/\sqrt{\rho }.$$

This can be used to calculate a volume-size-spectrum. Converted back to aerodynamic diameter (AD), the respective size channels are summed up and multiplied with the density ρ to get a mass concentration for the three PM${}_{x}$ size classes. Additionally, we computed the size class of so-called *Inhalable Coarse Particles* ${\mathrm{PM}}_{\left(10-2.5\right)}={\mathrm{PM}}_{10}-{\mathrm{PM}}_{2.5}$ since—as described above—we focused on detecting the larger particles and suspected that with the employed magnification, our images may not display scatter traces from the smaller particles.

The five sensors (dubbed *B001* to *B005*) had been 3D printed and manually assembled, all of them identically constructed as described above. Each of them was attached to a Samsung Galaxy S6 smartphone with at least 32 GB internal storage capacity running Android version 7.0. In terms of video recording options, the devices were configured slightly different to test different sensitivity (ISO) settings. In addition, three of the sensors were vented using an externally powered *SUNON UF383-100* microfan running at a voltage of 1.8 V and two were not vented in order to compare the detection performance. The settings for the five sensors are listed in Table 1.

The recorded videos were first processed with the CDPC algorithm described in the previous section. An algorithm parameter sweep was performed in which the following parameters were varied:

- MOG2 learning rates $r_{learn}$ of 0.1, 0.01, and 0.001
- number of standard deviations for Gaussian blur $\langle \sigma_{blur}\rangle$ of 5 and 9
- threshold for binarization $\theta_{b/w}$ of 10, 50 and 100
- threshold for contour detection $\theta_{contour}$ of 10, 50, 100, and 500

When analyzing the recorded images, it quickly became apparent that sensors *B002* and *B004* did not yield useful scatter traces at all, probably due to the lower ISO sensitivity. We therefore disregard them in our further analysis. While for each of the other three sensors, the videos showed clear scatter traces that were visible to the naked eye, initially we could not find a clear correlation with either of the three size classes PM${}_{10}$, PM${}_{2.5}$ or PM${}_{1}$. Instead, we observed a phase of high particle counts at the beginning of each of our measurement runs, that then quickly faded in spite of the fact that the PM${}_{x}$ concentration levels continued to rise.

As we already had suspected before, we assumed that we could only see the scatter traces from the fraction of the larger particles of the size spectrum, due to the employed magnification and our imperfectly constructed measurement chambers. This assumption was supported by the fact that the scatter traces were most dense during ca. 15 min after injecting the ammoniumsulfate into the chamber. Larger particles settle much faster than smaller ones and therefore could not be detected anymore afterwards. When we looked closer into the particle size distribution in the chamber over time, this assumption was substantiated by the data from the reference instrument: In the respective timeframe, particles up to ca. 4 μm were present in the chamber, while later dropping to a maximum of ca. 1 μm.

**Figure 8:**
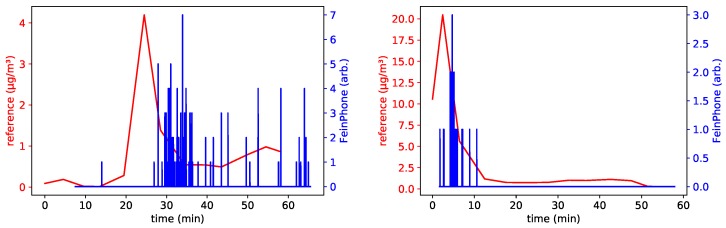
Particle counts obtained from the CDPC algorithm vs. reference signal (PM${}_{\left(10-2.5\right)}$ size fraction) for sensors B005 (**left**) and B001 (**right**). CDPC parameters: $r_{learn}$: 0.1, $\langle \sigma_{blur}\rangle$: 9, $\theta_{b/w}$: 50.0, $\theta_{contour}$: 100.0.

**Figure 9:**
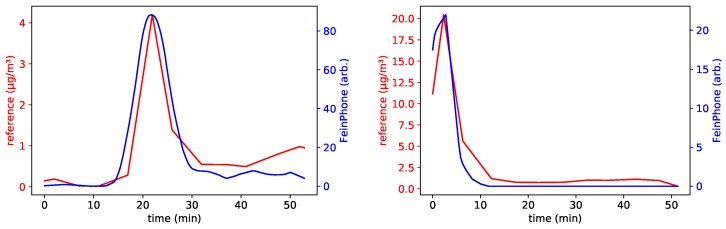
Combined approach: The output from the particle counting (CDPC) algorithm is subsequently piped through the Poisson Particle Detection (PPD). The graphs were shifted to compensate for the time delay caused by the reference method (see above). They show very good qualitative agreement with the PM${}_{\left(10-2.5\right)}$ size fraction of the reference, here shown for sensors B005 (**left**) and B001 (**right**).

This was also reflected in the FeinPhone readings. As a matter of fact, comparing the results of our analysis to the size fraction of coarse inhalable particles revealed an excellent qualitative correlation for the three sensors with high ISO settings (*B001*, *B003* and *B005*). Figure 8 shows the reference for PM${}_{\left(10-2.5\right)}$ and the particle counts obtained from analyzing the recorded videos using the CDPC algorithm. Figure 9 shows the same, but after additionally feeding the output of the CDPC algortihm into the PPD.

**Figure 10:**
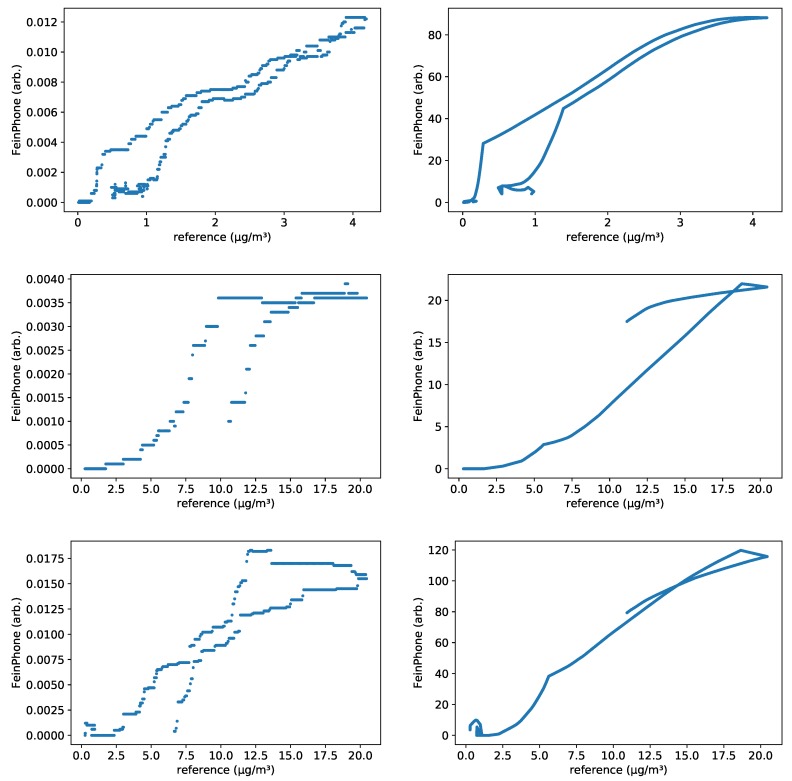
Scatterplots of the reference measurements vs. the results of the CDPC algorithm (**left**) respectively the combined CDPC/PPD algorithm (**right**), each for sensor *B005* (**top row**), *B001* (**middle row**) and *B003* (**bottom row**).

In order to quantify the obvious qualitative agreement, we calculated the *Pearson* correlation coefficient *r* for the individual datasets. The scatter plots in Figure 10 show a linear dependence between the *FeinPhone* signal and the reference measurement. The corresponding correlation coefficients are $r_{1}=0.969$, $r_{2}=0.968$, $r_{3}=0.954$, $r_{4}=0.975$, $r_{5}=0.987$ and $r_{6}=0.944$. The index corresponds to the enumeration of the plots from left to right and top to bottom.

In order to calculate the coefficients, we first corrected for the time shift between the *FeinPhone* and the reference data, and interpolated the signals in order to match them temporally. The plots were obtained in a three step process:

1. shifting
2. smoothing
3. interpolating

First, the reference signal was shifted by some fixed offset T≤9min. This corresponds to a compensation for the sampling delay of the reference device. As explained above, due to the channel scanning measurement principle of the reference device, a rising concentration within the measurement interval can be delayed in the output by up to 9 min. In the two measurement sessions we conducted, the time shift offsets were ≈3min respectively ≈7.5min. The time shift was consistent for all sensors within each of the sessions.

Second, the *FeinPhone* signal was smoothed by a moving average with window size of 10,000 points. For a sampling rate of 30 fps, this corresponds to a time interval of ≈5.5min. This is a reasonable choice based on the reference sampling interval of 4.5 min.

Third, the shifted reference and smoothed *FeinPhone* data were linearly interpolated to allow for exact temporal matching of scatter points. Within the overlap region of both signals, the interpolations are evaluated every second to generate scatter points.

All correlation coefficients are in good agreement with the hypothesis of linear dependence. Indeed, this is also what one would physically expect since the mass concentration of fine dust is proportional to the number of particles, if all particles share the same size. This can be seen as a rough approximation of the physical reality, because we analyzed PM${}_{\left(10-2.5\right)}$ and therefore particles of approximately homogeneous size.

## 6. Discussion

This section discusses some of the limitations and possible future improvements of the prototype sensor.

### 6.1. Ventilated vs. Unventilated

**Figure 11:**
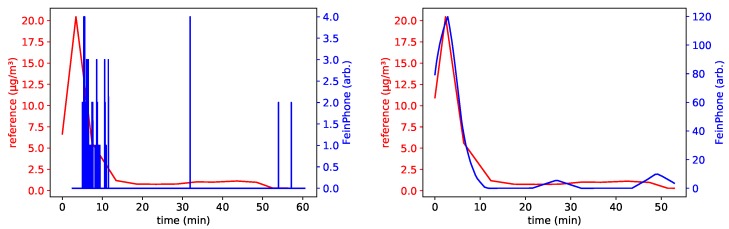
Results from the unventilated sensor (*B003*). In our lab experiments, it did not make a notable difference whether the sensor was ventilated or not (cmp. data of sensor *B001* in Figure 8 respectively Figure 9).

Another aspect of our evaluation concerns the sampling of the air, i.e., whether the measurement chamber was actively vented by a fan or whether we relied on diffusion to transport particles into the sensor. Both the ventilated (Figure 9) and the unventialated version (Figure 11) of our sensor performed similarly.

However, whether this result can be transferred to real-life measurements remains unclear and needs to be properly evaluated. In the lab environment, the aluminum measurement box itself was ventilated, possibly facilitating quicker air exchange in the passive sensors as well.

Proper ventilation of the measurement chamber to ensure that individual measurements are actually independent may also be an issue. An approach to make a fan-less measurement system could be to vent the detector volume by moving the phone and use the phone’s inertial sensors to estimate the flow rate through the measurement chamber.

### 6.2. Detection Size Limit

In our evaluation, the CDPC algorithm was only successful in detecting coarser particles. After these had settled, no individual counts were detected anymore. This is, as discussed above, a tradeoff between the employed magnification and the size of the measurement volume that can be sampled.

Additionally, outdoors this may be different. As naturally occurring turbulence keeps the coarser size fraction suspended for a longer period of time (or resorbs particles), this limitation may be less relevant.

**Figure 12:**
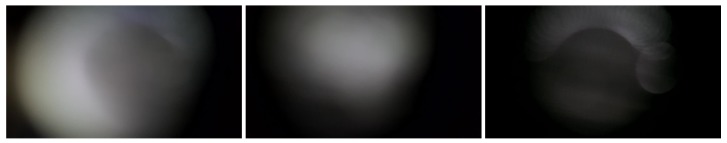
Due to rapid prototyping with a 3D printer and the manual assembly of the sensor hardware, the measurement chambers were not 100% identical, resulting in different background illumination.

Finally, part of the detection size limit may be a hardware issue. Due to the imperfect measurement chambers that were a result of rapid prototyping of the sensor, we may have lost information. All of our sensors exhibited different backgrounds due to 3D printing and manufacturing of the sensor modules (see Figure 12).

The background subtraction step of the CDPC algorithm turned out to remove some faint particle traces that could be identified by humans in the original recordings and are subtracted along with the background image. A possibility would be to fine-tune the algorithm to each individual sensor case. However, since the background images vary, this would require a calibration step for each sensor. In addition, background illumination from scattered particles that are too small to have shown as individual traces could still be measurable, were it not for the background illumination of the imperfect sensors. A much more sensible approach would therefore be to first perfect the light trap design, so that no background image is visible in the absence of particles. This can be achieved with a thorough optical design, which was not the focus of this paper. The construction material of the chamber and its surface can also be improved from the 3D printed versions. Additionally, the inside of the light trap could be coated with absorbing paint.

### 6.3. Computational Requirements

The data analysis for this work was not done online on a smartphone, but rather after the data recording phase on a desktop computer, in order to test different parameter settings of the algorithms. However, in the Android application we designed for recording, both data storage as well as (near) real-time processing of the images is possible, depending on image resolution and recording framerate. Naturally, the lower the image resolution, the faster the processing is, and therefore the lower are the requirements for storage of the raw data. As discussed in the previous section, a better light trap could most likely eliminate the need for background subtraction and thus reduce the processing complexity greatly.

## 7. Conclusions

This paper presented *FeinPhone*, a phone-based fine dust measurement system that uses the camera and flash LED that are available on off-the-shelf smartphones as light receptor and emitter of a passive clip-on dust sensor respectively. The system realizes a novel counting approach based on light-scattering particle sensors which leverages the additional information gained by having a 2D sensor (the camera) instead of a single photodiode. This allows capturing the scatter traces from individual particles rather than just retaining a light intensity sum signal as in simple photometers.

Our evaluation comparing several *FeinPhone* sensors with data from a high-quality APS/SMPS reference device at the World Calibration Center for Aerosol Physics shows that the collected data shows excellent correlation with the inhalable coarse fraction of fine dust particles (r > 0.9) and can successfully capture its levels under realistic conditions.

In future work, we intend to extend this novel counting approach beyond the proof-of-concept shown in this paper. For this, a more sophisticated light trap design is advisable first and foremost, to reduce the information loss by the background subtraction step and potentially extend the detection capabilities to the full particle size spectrum.

## Figures and Tables

**Table 1:** Device configuration in CDPC evaluation.

Sensor	Fan	ISO	Shutter time	Framerate	Focal length setting	Resolution
*B001*	yes	400	33.33 m s	30	10 (inf)	1920 × 1080
*B002*	yes	200	33.33 m s	30	10 (inf)	1920 × 1080
*B003*	no	400	33.33 m s	30	10 (inf)	1920 × 1080
*B004*	no	200	33.33 m s	30	10 (inf)	1920 × 1080
*B005*	yes	400	33.33 m s	30	10 (inf)	1920 × 1080

## Abbreviations

The following abbreviations are used in this manuscript:

ABS	Acrylonitrile Butadiene Styrene
AD	Aerodynamic Diameter
APS	Aerodynamic Particle Sizer
BC	Black Carbon
CCD	Charge-Coupled Device
CDPC	Comtour Detection Particle Counting
CMOS	Complementary Metal–Oxide–Semiconductor
ISO	Camera exposure index rating
LED	Light Emitting Diode
MEMS	Microelectromechanical systems
PLA	Polylactic Acid
PM	Particulate Matter
PPD	Poisson Particle Detection
RGB	Red, Green, Blue (color model)
SMPS	Scanning Mobility Particle Sizer
SPEX	Spectropolarimeter for Planetary Exploration
SPP	Spatial Poisson Process
WCCAP	World Calibration Center for Aerosol Physics
WMO	World Meteorological Organization
